# Dr. G. P. Howe of Boston, Mass.

**Published:** 1917-11

**Authors:** 


**Dr. G. P. Howe of Boston, Mass.,** was killed in action, Sept. 28, while on duty with the British forces in France. He is the second member of the Medical Reserve Corps to have died from strictly military casualties. We understand that no officers outside the Medical Corps have died of wounds and that the deaths from hostilities have been limited to this corps, though several officers and privates of the line have died of disease or accident, including those due to practice with bombs and fire arms in France, or from strictly military risks, as with aeroplanes and from attack while on guard [illegible]ty in this country. Since Sept 18, up to Oct 20, deaths in the M. R. C. number 3, all in men on duty at camps, one due to cutting throat, probably suicide, 2 to drowning, one of the latter in a gallant attempt at rescuing another.

